# A homozygous *LAMB3* frameshift variant in junctional epidermolysis bullosa-affected Bleu du Maine sheep

**DOI:** 10.1007/s13353-025-00957-5

**Published:** 2025-03-18

**Authors:** Anna Letko, Liesbeth Harkema, Karianne Peterson, Reinie Dijkman, Cord Drögemüller

**Affiliations:** 1 Institute of Genetics, Vetsuisse Faculty, University of Bern, 3012 Bern, Switzerland; 2 Department of Pathology, Royal Animal Health Services (GD), P.O. Box 9, 7400 AA Deventer, The Netherlands; 3 Department of Small Ruminant Health, Royal Animal Health Services (GD), P.O. Box 9, 7400 AA Deventer, The Netherlands

**Keywords:** *Ovis aries*, Genodermatosis, Whole-genome sequencing, Skin fragility, Laminin

## Abstract

Epidermolysis bullosa (EB) is a group of inherited skin disorders characterized by skin fragility and blistering. Here, four Bleu du Maine lambs, sired by one ram, were diagnosed with EB very early in life. Due to the severity of the clinical signs, the lambs had to be euthanized. The affected lambs exhibited hoof sloughing and multiple ulcerations on the head, oral cavity, skin over the joints, and the ruminal pillars. Histopathology showed abrupt subepidermal clefts, epidermal detachment directly above the basal membrane, and ulcerations consistent with junctional EB (JEB). Two cases underwent whole-genome sequencing (WGS) to identify the genetic cause. Genomic analyses with the hypothesis of autosomal recessive inheritance identified the most likely pathogenic homozygous 1-bp deletion in the *LAMB3* gene (NC_056065.1:g.73166198delG). Recessive forms of JEB in humans and dogs are caused by variants in *LAMB3* gene, which encodes β3 subunit of laminin 332, a critical component of the epidermal basal membrane. The ovine frameshift variant putatively introduces a premature stop codon and disrupts the donor splice site of exon 20. The variant allele was homozygous in both sequenced cases and heterozygous in three unaffected close relatives and was absent in 1075 unrelated control sheep of various other breeds. This study highlights the importance of genetic investigation in veterinary diagnostics of and represents the first report of a *LAMB3*-related recessive EB in sheep. The findings enable genetic testing to inform breeding strategies and provide a second spontaneous large animal model for *LAMB3*-related JEB in humans.

**Supplementary Information:**

The online version contains supplementary material available at 10.1007/s13353-025-00957-5.

## Introduction

Epidermolysis bullosa (EB) represents a group of inherited skin disorders characterized by the skin’s and mucous membranes’ fragility, resulting in blistering and erosions in response to trauma or friction (Mauldin and Peters-Kennedy 2016). It has been extensively studied in humans as well as in domestic animals, shedding light on common pathophysiological mechanisms and potential implications for veterinary medicine (Leeb et al. 2017).

EB is caused by variants in genes encoding various structural proteins at the level of the basal keratinocytes and basal membrane zone, such as keratins, collagens, and laminins (Mauldin and Peters-Kennedy 2016). Classical EB in humans is classified into four types based on the level of detachment and the structural proteins involved: EB simplex (EBS, OMIM:PS131760), junctional EB (JEB, OMIM:PS226650), dystrophic EB (DEB, OMIM:120,120), and Kindler EB (KEB, OMIM:173,650). EBS may be inherited as an autosomal dominant or recessive trait and is characterized by defects in basal cell keratins resulting in basal cell cytolysis and intraepidermal clefts. Defects in hemidesmosome-associated proteins cause JEB, an autosomal recessive disease, that leads to clefts in the lamina lucida. DEB is caused by defects in the anchoring fibrils with clefts in the superficial dermis below the lamina densa of the basal membrane and has dominant and recessive subtypes (Mauldin and Peters-Kennedy 2016). KEB shows a mixed level of skin cleavage and an autosomal recessive mode of inheritance (Has et al. 2020).

This genodermatosis has been also studied in veterinary medicine and the classical EB types were found to be largely homologous between humans and various domestic animals (Leeb et al. 2022; Nicholas et al. 2024). In sheep, only two variants in *ITGB4* (Suárez-Vega et al. 2015; Fabre et al. 2021) and one in *LAMC2* (Mömke et al. 2011) genes were described in JEB-affected sheep to date (OMIA:001948–9940, OMIA:001678–9940). Lack of collagen VII, without investigation of the underlying molecular genetics, was reported in DEB-affected Swiss White Alpine sheep (OMIA:000341–9940) (Bruckner-Tuderman et al. 1991). A comparative summary and schematic representation of different epidermolysis bullosa forms is summarized in supplementary file S1 (Online resource 1).

The severity of EB in animals is variable, reflecting the heterogeneity observed in human patients. The spectrum of severity largely correlates with the type of variant and its consequences at the mRNA and protein level, with most intermediate forms being associated with missense variants, whereas severe forms are more often caused by loss-of-function variants that result in premature stop codons and out-of-frame transcripts that are either eliminated by nonsense-mediated decay or translated into truncated proteins (Nakano et al. 2002). However, the genotype–phenotype correlation may be more difficult to predict for splice site variants (Wen et al. 2024).

This report aims to elucidate the genetic etiology of this debilitating disorder in order to improve the overall management and welfare of affected Bleu du Maine sheep flocks. Affected animals display signs of pain, discomfort, and impaired skin barrier function, making their welfare a major concern, particularly for livestock producers. In addition, understanding naturally occurring forms of EB in animals offers valuable insights into potential therapeutic strategies in both veterinary and human medicine (Medeiros and Riet‐Correa 2015).

## Material and methods

Over the course of two lambing seasons, four Bleu du Maine lambs on three farms were reported with severe skin lesions. Lambs of both sexes were affected, and their age ranged from 6 weeks to 2 months. All lambs were reportedly sired by the same ram that was unfortunately not available for sampling. Three of the affected lambs were euthanized and submitted for pathological examination. Tissue samples of the lesional skin, claws, tongue, and rumen were collected for routine histologic examination using the hematoxylin and eosin (H&E) staining. Periodic acid-Schiff stain (PAS) was used to visualize the basal membrane.

For genetic diagnostics, EDTA-blood samples were collected from two of the histologically confirmed EB-affected lambs, their two dams and one apparently normal full sibling of an affected lamb. All five animals were found to be negative for the two known JEB-associated ovine recessive variants in *LAMC2* (Mömke et al. 2011) and *ITGB4* (Fabre et al. 2021).

Subsequently, the genomic DNA of two EB-affected lambs was whole-genome sequenced (WGS) on an Illumina NovaSeq6000 instrument using a PCR-free fragment library resulting in an average read depth of 14 × . The reads were mapped and variants were called using the GATK pipeline (Van der Auwera and O’Connor 2020) with respect to the ovine reference ARS-UI_Ramb_v2.0 (GCF_016772045.1) as described in detail previously (Letko et al. 2023). Plink v1.9 (Chang et al. 2015) was used to validate the sex assignment and relatedness of the lambs. Additionally, variant prioritization was conducted using publicly available WGS data of 116 unrelated control sheep (Online resource 2). Furthermore, the allele frequency of detected variants was determined in 935 genomes representing 70 different breeds from the Sheep Genomes Project Variant Database (SGPVD) (Daetwyler et al. 2019).

Two in silico effect prediction tools, PredictSNP (Bendl et al. 2014) and MutPred2 (Pejaver et al. 2017), were used to evaluate the predicted impact of the candidate variants on the resulting proteins. Additionally, the SpliceAI web server (Broad Institute 2020) was used for the prediction of the candidate variant’s impact on splicing (Jaganathan et al. 2019). The final candidate causal variant in the *LAMB3* gene was validated and genotyped in 24 further animals from unrelated breeds (Engadine red, Border Leicester, and Swiss black-brown mountain sheep) available from the Vetsuisse Biobank by Sanger sequencing of PCR amplicons using the following primers: forward: 5′-GAGAGCACCTTCACTCAGAGC-3′ and reverse: 5′-TGAGTGTGCAGGTCCATTCCC-3′.

## Results and discussion

A 2-month-old lamb from a flock of purebred Bleu du Maine sheep was presented with skin lesions and lameness with claw detachment. During the subsequent lambing season, analogous lesions were observed in three Bleu du Maine lambs from two different flocks. Given the severity of the clinical signs, it was necessary to humanely euthanize all affected lambs. Considering the pathological lesions and the young age of the lambs, a presumptive diagnosis of EB was made. Pathologic examination was conducted on three of the lambs and showed identical histopathologic skin lesions in all cases.

**Fig. 1:**
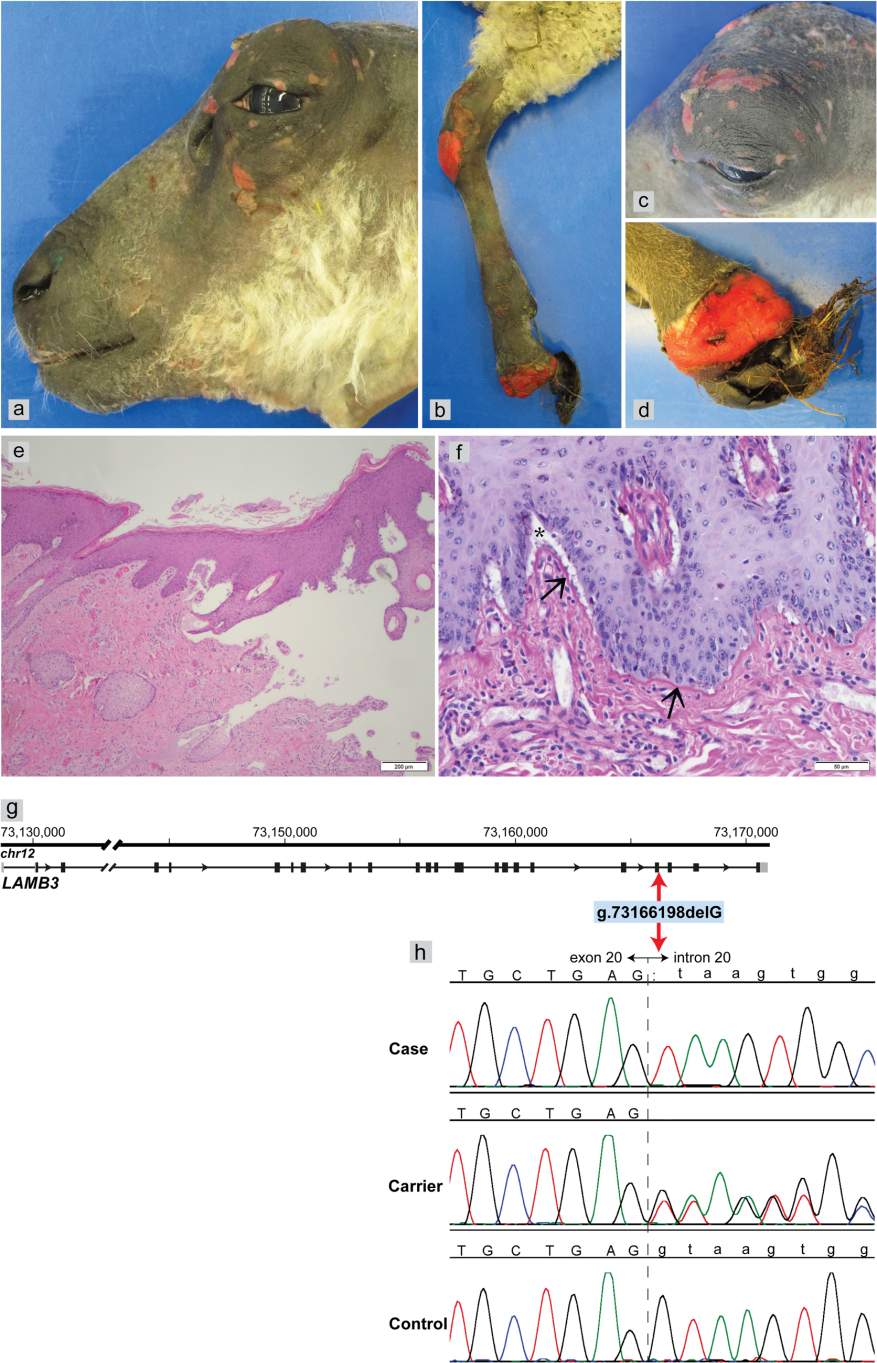
Phenotype and genotype analyses in a junctional epidermolysis bullosa-affected Bleu du Maine lamb. Sharply demarcated ulcers were present over bony prominences and sites exposed to friction around the eyes and on the head (**a**, **c**) and on the carpal joints (**b**). Sloughing of the claws affected all feet (**b**, **d**). Histopathologically, in the H&E stain, there was a large cleft between the epidermis and the dermis with minimal inflammation in the dermis (**e**). The PAS stain on higher magnification (**f**) visualized subepidermal clefts (indicated by asterisks (*)) above the PAS-positive basal membrane (arrows). Genetic investigation revealed a homozygous 1-bp deletion in the *LAMB3* gene (**g**) that putatively alters the donor splice site of exon 20 (**h**)

Upon macroscopic examination, the lambs showed hoof sloughing and sharply delineated skin ulcers on the head around the eyes, on the dorsal side of both carpal joints, and on the plantar sites of the tarsal joints (Fig. 1a–d). Additionally, ulcers were observed in the oral cavity on the tongue and on rumen pillars. Histologically, the lesions were characterized by abrupt subepidermal clefts and ulceration, either with minimal inflammation or superficial secondary fibrinopurulent inflammation (Fig. 1e). An additional PAS stain demonstrated clefts above the PAS-positive basal membrane (Fig. 1f).

The PAS-positive basal membrane was localized at the base of the cleft, indicating either EBS or JEB in these lambs. However, further morphological differentiation by ultrastructural examination or basal membrane antigen mapping was not feasible. Very subtle derangements in the basal keratinocytes have been observed, which could be indicative of EBS; however, the evidence was considered insufficient for reliable differentiation between the various types of EB.

All lambs diagnosed with EB were sired by the same ram and all parents, as well as other lambs in the flocks, were apparently unaffected. Therefore, a single rare breed-specific allele with autosomal recessive mode of inheritance was hypothesized as the most probable underlying genetic cause. WGS of two EB-affected lambs was carried out and the proportional identity-by-descent value was found to be 0.59, thereby confirming their close relatedness. Inspection of the genomic variants for shared homozygous SNVs and indels revealed that both animals were homozygous for the alternative allele at 2825 autosomal loci, while all 116 control sheep of various other breeds were homozygous for the reference alleles at these loci. Of the shared variants, 1099 were detected in the global cohort of 935 sheep (Daetwyler et al. 2019), resulting in a list of 1726 private variants, of which only 23 were predicted to be protein-changing: 1 frameshift and 22 missense variants (Online resource 3). A single missense variant in the *GPR45* gene (chr3:g.97281474G > A) was predicted deleterious by one of the in-silico prediction tools. This gene encodes a member of the G protein-coupled receptor family, functions in the central nervous system, and is not known to be associated with any skin disease (Safran et al. 2021; GeneCards 2024).

The only high-impact variant was identified in the *LAMB3* gene (Fig. 1g) and was predicted to result in a frameshift and premature stop codon (chr12:g.73166198delG; c.3051 + 1delG; p.(Val1018PhefsTer12)). According to the 3'-rule of the HGVS Nomenclature (den Dunnen et al. 2016), the 1-bp deletion affects the first nucleotide of intron 20 at the exon/intron boundary (Fig. 1h), potentially leading to altered splicing of exon 20 ((XM_042229255.1):r.spl). The SpliceAI results indicated that the deletion would disrupt the donor splice site of intron 20 with a probability of 100% and predicted a cryptic donor splice site 1 bp upstream with a probability of 72%. In both scenarios, the frameshift introduces a premature stop codon truncating 12.2% of the wildtype LAMB3 protein. As the animals were euthanized at the time of genomic investigation, an experimental analysis of the RNA was not possible and thus the consequences at the transcript level could not be precisely validated. Sanger sequencing was used to confirm the *LAMB3* variant (Fig. 1h) and to genotype the close relatives of the affected lambs and unrelated control sheep of other breeds (Table 1).

**Table 1:** Genotype distribution of the *LAMB3*:c.3051 + 1delG variant in the JEB-affected Bleu du Maine sheep, their unaffected dams and a sibling, and control sheep of unrelated breeds

*LAMB3* genotype	ref/ref	ref/del	del/del
Bleu du Maine sheep (*n* = 5)	0	3	2
Population control sheep* (*n* = 1075)	1075	0	0

*The control cohort included 24 sheep genotyped by PCR, 116 in-house generated whole-genome sequences, and 935 whole-genome sequences from the public SGPVD global cohort

The *LAMB3* gene is a functional candidate gene for JEB as it encodes the β3 chain, which together with α3 and γ2 chains form a heterotrimeric protein laminin 332, a critical component of the epidermal basal membrane a critical component of the epidermal basal membrane (Domogatskaya et al. 2012). In humans, various recessive variants in the three laminin genes (*LAMA3*, *LAMB3*, *LAMC2*) have been described in patients with JEB (OMIM:PS226650). It has been demonstrated that more than a fifth of disease-causing variants in JEB are splicing variants that lead to exon skipping or cryptic splice site activation, which contributes to the explanation of disease severity (Wen et al. 2024). In veterinary medicine, all three laminin genes have also been associated with forms of JEB (OMIA:001677, OMIA:002269, OMIA:001678). Variants in *LAMA3* have been previously reported in JEB-affected dogs (Capt et al. 2005; Herrmann et al. 2021), horses (Graves et al. 2009), and cattle (Sartelet et al. 2015). So far, only one missense variant in canine *LAMB3* has been described in dogs diagnosed with an intermediate form of JEB (Kiener et al. 2020). *LAMB3* was also identified as a candidate gene in a single JEB-affected cat, however, at that time no molecular genetic investigation was conducted (Alhaidari et al. 2005). In sheep, a *LAMC2*-related severe form of JEB is known in German Black Headed Mutton, caused by a 2-bp frameshift deletion that was shown to create a premature stop codon and introduce an alternative splice site (Mömke et al. 2011). Furthermore, *LAMC2* also harbors pathogenic variants associated with JEB in horses (Spirito et al. 2002) and cattle (Murgiano et al. 2015).

In conclusion, the genomic analyses of two related JEB-affected lambs identified a single homozygous private protein-changing variant in *LAMB3*, a known candidate gene for JEB in humans and dogs. In light of the histopathological and genomic findings, it can be postulated that this variant causes a recessive form of JEB in the Bleu du Maine sheep. The findings provide a spontaneous large animal model for potential JEB treatment development and support a more precise diagnosis of ovine EB forms. Moreover, a genetic test should be employed to screen the breeding population with the objective of estimating the variant allele frequency and to avoid at-risk matings that could result in further affected animals.

## Supplementary Information

Below is the link to the electronic supplementary material.

ESM 1 (587 KB)

ESM 2 (16.3 KB)

ESM 3 (222 KB)

## Data Availability

All whole-genome sequences associated with this study are deposited at the European Nucleotide Archive (https://www.ebi.ac.uk/ena/), and all study and sample accession numbers are provided in Table S1 (Online resource 2). The SGPVD data of 935 sheep is available from the CSIRO Data Access Portal: https://doi.org/10.25919/5d39e494936c6.
