# Supplementary material for: A homozygous LAMB3 frameshift variant in junctional epidermolysis bullosa-affected Bleu du Maine sheep
Source: J Appl Genet. 2025 Mar 18;66(3):709–14. doi: 10.1007/s13353-025-00957-5 (PMC12367931; doi:10.1007/s13353-025-00957-5)
Supplement: Supplementary file 1 — (587 KB) [file 13353_2025_957_MOESM1_ESM.docx]

|  | 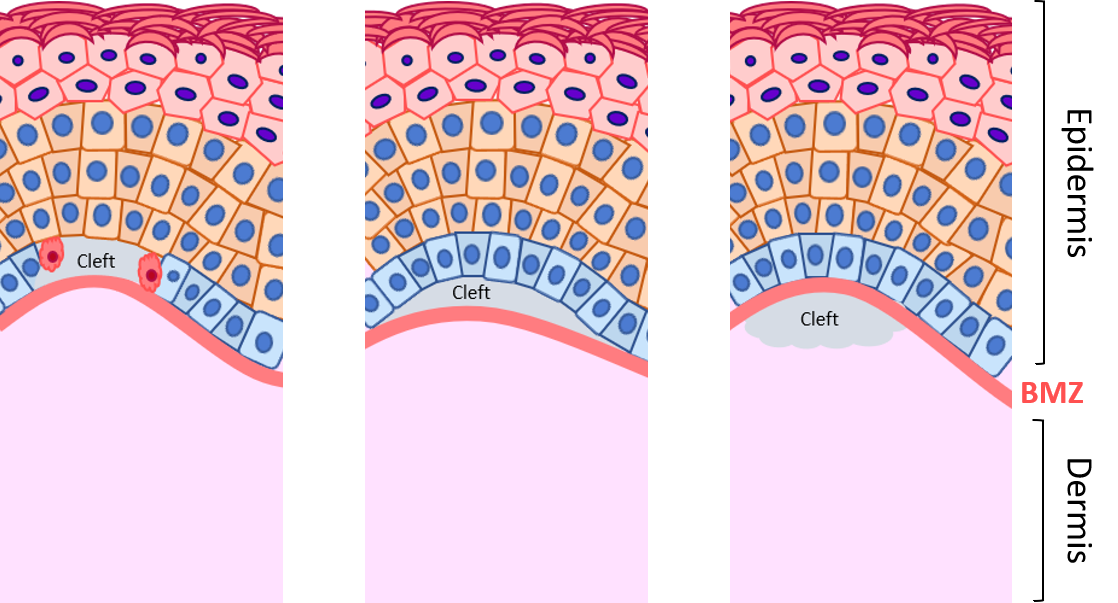**Epidermolysis bullosa subtypes** | | | |
| --- | --- | --- | --- | --- |
|  | **Epidermolysis bullosa simplex** | **Junctional**  **epidermolysis bullosa** | **Dystrophic**  **epidermolysis bullosa** |  |
| **Associated genes** | Defects in cytoplasmic cytoskeleton filaments  Keratins:  - *KRT5* (cattle, dog)  - *KRT14* (cat)  Plectin:  - *PLEC* (dog)  Undetermined gene (buffalo, cattle, dog) | Defects in hemidesmosomal proteins  Severe generalized EB (formerly Herlitz type):  Laminin 332 coding genes:  - *LAMA3* (cattle, dog, horse)  - *LAMB3* (cat, dog, sheep)  - *LAMC2* (cattle, horse, sheep)  Undetermined gene  (cattle, dog, horse, sheep, mouse, rat)  Intermediate generalized (formerly non-Herlitz type):  Laminin 332 coding genes:  - *LAMA3*, *LAMB3*, *LAMC2*  Collagen XVII:  - *COL17A1* (cat)  Integrins α6 and β4:  - *ITGA6* (cattle) - *ITGB4* (cattle, sheep)  Undetermined gene (cat, dog) | Defects in anchoring fibrils  Collagen VII:  - *COL7A1* (cattle, dog, sheep)  Undetermined gene  (cat, cattle, dog, goat, sheep, ostrich) |  |
| **Separation site** | Cleft formation in the basal layer, with or without basal cytolysis | Cleft formation in the lamina lucida of the basement membrane zone (BMZ) | Cleft formation in the superficial dermis (Sublamina densa) |  |
| **Histopathology** | PAS positive basement membrane at the bottom of the cleft | PAS positive basement membrane at the bottom of the cleft | PAS positive basement membrane at the roof of the cleft |  |
| **Clinical and pathological findings** | Onset of lesions in the first week of life.  Skin blistering and ulceration on pressure points, glabrous areas, and limbs, ulcers in the oral cavity, hoof/claw sloughing.  Growth retardation. | Onset of lesions at birth.  Skin blistering and ulceration more generalized, ulcers in the oral cavity, ruminal ulcers, anal ulcers, vaginal/vulvar ulcers.  Growth retardation.  The severe form (Herlitz type) is usually lethal within the first week. | Onset of lesions in the first week of life.  Skin blistering and ulceration on pressure points, glabrous areas, and limbs, ulcers in the oral cavity, esophagus. and vulva. Hoof/claw sloughing.  Healing with scar formation is possible.  Growth retardation. |  |

**File S1. Comparative summary and schematic representation of different epidermolysis bullosa forms**

*Adapted from Medeiros GX and Riet-Correa F (2015)* *Epidermolysis bullosa in animals: a review. Vet Dermatol, 26:3.* [*https://doi.org/10.1111/vde.12176*](https://doi.org/10.1111/vde.12176)
